# Postnatal care service utilization and its determinants in East Gojjam Zone, Northwest Ethiopia: A mixed-method study

**DOI:** 10.1371/journal.pone.0256176

**Published:** 2021-08-17

**Authors:** Liknaw Bewket Zeleke, Asmare Talie Wondie, Melaku Admas Tibebu, Addisu Alehegn Alemu, Mekuanint Taddele Tessema, Nigusie Gashaye Shita, Marjan Khajehei

**Affiliations:** 1 College of Health Sciences, Debre Markos University, Debre Markos, Ethiopia; 2 College of Natural and Computational Sciences, Debre Markos University, Debre Markos, Ethiopia; 3 Women’s and Newborn Health, Westmead Hospital, Westmead, New South Wales, Australia; 4 Westmead Clinical School, University of Sydney, Sydney, Australia; 5 School of Women’s and Children’s Health, University of New South Wales, Sydney, New South Wales, Australia; University of Mississippi Medical Center, UNITED STATES

## Abstract

**Background:**

The days and weeks after childbirth are crucial for both the mother and her newborn child leading for the majority of maternal and perinatal mortalities. The World Health Organization recommends at least three postnatal visits within 42 days after birth. However, postnatal care utilization remains low worldwide. Quantitative findings revealed low utilization of postnatal care in Ethiopia, however, no study explored the barriers for postnatal care. This study aimed to assess the barriers to postnatal care service utilization in East Gojjam Zone, Northwest Ethiopia.

**Methods:**

A community-based, mixed type cross-sectional study was conducted from December 15, 2018, to February 15, 2019. The quantitative data was gathered using the interviewer-administered interviewing technique from 751 women who gave birth within one year prior to the study selected by multistage sampling. The qualitative data were collected from purposively sampled women, facility leaders, and health extension workers using in-depth interviews and focused group discussions. The quantitative and qualitative data were analyzed using logistic regression and by the thematic content analysis method, respectively.

**Results:**

The study revealed that postnatal care service utilization was 34.6%. The odds of using PNC services were greater in women aged 25–34 years and used maternity waiting home. In contrast, women who were muslim religion followers, had normal or instrumental birth, not aware of the PNC services and whose partners were not supportive of the use of MCH services were less likely to use PNC services. According to the qualitative findings, lack of awareness, traditional beliefs and religious practices, distance and transportation, environmental exposure, and waiting time were identified as barriers to PNC service utilization.

**Conclusion and recommendation:**

The study showed low utilization of PNC services in East Gojjam zone, northwest Ethiopia. Improvements in personal health education, in construction of relevant infrastructure, and to transport, are needed to remove or reduce barriers to PNC service use in East Gojjam Zone, Northwest Ethiopia.

## Background

The days and weeks after birth are critical for a woman and her newborn child as their risk of developing long-term health problems is increased during this period. This postnatal period is also a period of adaptation to multiple changes, physiologically, socially and psychologically [1].

The term postnatal refers to all issues relating to the mother and child after birth, whereas the postnatal period is defined as starting immediately after the birth and for the first six weeks of life. Hence, postnatal care (PNC) services are services provided to new mothers within the first six weeks [2].

The World Health Organization (WHO) suggests that a woman and her infant to have postnatal visits at least three times within the first six weeks after birth. The first should occur within 48–72 hours after birth (irrespective of the place of childbirth), the second between seven and 14 days, and the third at six weeks. The American College of Obstetricians and Gynecologists (ACOG) also recommends that postpartum care be continual, with services individually tailored to each woman. ACOG considers this care to be a ‘fourth trimester’ service [2–4].

Failure to get the PNC within 42 days after birth may result in death and/or disability as well as missed opportunities to promote healthy behaviors, affecting women, newborns, and children. Globally, nearly two-thirds of maternal deaths and nearly half of perinatal mortalities occur within the days and weeks after birth, and in Africa two-thirds of newborn deaths are avoidable. A local study in Ethiopia also revealed that nearly three-quarters of maternal deaths occur during the postpartum period [5–8]. Despite this knowledge, postnatal care utilization remains low worldwide. Globally, less than one-third of mothers return for postnatal care after giving birth, according to the WHO report on PNC utilization. In developing countries, especially in sub-Saharan Africa, the number of women requesting, and receiving, postnatal care remains critically low. According to the 2016 Ethiopia Demographic and Health Survey, postnatal care service utilization was alarmingly low, with only one in five women visiting health institutions for postnatal care services [9]. A cross-sectional study revealed that only one-third of new mothers seek PNC services after giving birth in the town of Debre Markos [10–13]. The provision of timely, and high quality, PNC services could save 67% of newborn deaths in sub-Saharan Africa [14].

Different researchers have tried to quantify postnatal care service utilization and have uncovered some associated factors, but no qualitative study has explored the reasons for the low utilization of PNC services nationally, including the area in our study. Hence, our study aimed to quantitatively and qualitatively explore the barriers to PNC utilization in East Gojjam Zone, Northwest Ethiopia.

### Operational definitions

#### Postnatal period

Is a period that begins immediately after the birth of the baby and extends up to six weeks (42 days) after birth [2].

#### Postnatal care service utilization

Is the use of postnatal care services at least once in health facilities by women up to 42 days after childbirth, for a home birth, and post-discharge for those mothers who delivered in a health institution.

#### Maternity waiting homes

Are residential facilities, located near a qualified medical facility, where women defined as "high risk" can await their childbirth and be transferred to a nearby medical facility shortly before childbirth, or earlier should complications arise. In 2015, the WHO recommended the service to be offered to all pregnant women in their late weeks of gestation to prevent delays resulted from geographic barriers [15, 16].

## Methods

### Study area, period and design

The study was conducted in East Gojjam Zone, in the Amhara region of Northwest Ethiopia, from December 15, 2018, to February 15, 2019. Debre Markos is the capital city of East Gojjam Zone and is located around 297 km from Addis Abeba, the capital city of Ethiopia, and 220 km away from Bahir Dar, the capital city of the Amhara region. According to the 2007 Central Statistical Agency Report, the population of East Gojjam Zone is 2,153,937, half of whom are female. This zone has 18 districts, nine government hospitals, and 101 health centers. A community-based, mixed type of cross-sectional study design was used.

### Sample size determination and procedures

The sample size required for the quantitative aspect of the study was determined using a single population proportion formula with the following assumptions: the proportion of postnatal care utilization was 33.3% (p = 0.333) [13], level of significance 5% (α = 0.05), Z α/2 = 1.96, absolute precision or margin of error 5% (d = 0.05) and non-response rate 10%. Using the formula n = [Z α/2*p(1-p)]/d^2^ = 1.96^2^*0.333(1–0.333)/0.05^2^, the sample size n was calculated to be 341.3. Since the sampling method required the application of multistage sampling, a design effect of two was used. Finally, considering a 10% contingency rate for non-responses, the final sample size was calculated as 751.

The qualitative data were collected from women, health facility leaders, and health extension workers. Three focused group discussions (FGD) and three in-depth interviews with women, three one-on-one in-depth interviews with health facility leaders, and three in-depth interviews with health extension workers were conducted. The final sample size was mainly determined based on the information saturation level. Eight women participated in two FGDs and seven women took part in one FGD, hence the total number of participants was 23.

The participants of the quantitative study were selected using a multistage random sampling method that applies simple random sampling in different steps. Initially, five districts were randomly selected from East Gojjam Zone followed by the selection of five kebeles (the smallest local administrative structure) from each district. Finally, the required number of participants was selected from each kebele using the health extensions’ maternity registration book to constract the sampling frame. An equal number of participants was allocated at the district level and the population size proportion allocation technique was applied at the kebele level.

For the qualitative aspect of the study, participants for the in-depth interview and focused group discussions were selected using the purposive non-random sampling method. Mothers were selected during quantitative data collection. Facility leaders and health extension workers were interviewed at their offices.

### Data collection procedure

The quantitative and qualitative data were collected concurrently. Primary data were collected using a structured questionnaire and unstructured interviewer guide in the Amharic language. This questionnaire was initially developed in English after a thorough literature review and then translated to Amharic. Ten Bachelor of Science qualified midwives collected the data under the supervision of five Master of Science qualified midwives. The principal investigator and co-investigators were available on site to give guidance as needed and to check the data quality. Informed written consent was obtained from each participant and their privacy was maintained during data collection. Confidentiality was also guaranteed at the conclusion of the study and while disseminating the results by using deidentified data.

### Data quality control

The questionnaire and interviewer guide were prepared separately for each data collection method according to the nature of participants. Data collectors and supervisors were recruited and received training for two days. The questioning tool was pretested prior to the actual data collection to check its appropriateness and the familiarity of the data collectors with the tool. The data collectors gathered the data under continuous supervision and guidance by the supervisors and investigators. For the qualitative data, the audio file, consent form, field note, and FGD enrolment form were consistently coded.

### Data analysis

#### Quantitative data

The quantitative data were checked for completeness, inconsistencies, and missing values and then coded and entered into Epidata software version 4.2. Data cleaning and analysis were conducted using Statistical Package for Social Science (SPSS) version 25 and STATA software version 14 respectively. Descriptive statistics (mean, standard deviation, and percentage) were computed to describe the study population in relation to socio-demographic and other relevant variables. The association between each independent variable with postnatal care service utilization was determined by bivariate analysis and variables with a *p*-value <0.2 were entered to multivariable analysis. Finally, variables with a *p*-value of <0.05 were considered as significantly associated with postnatal care service utilization. The direction and strength of the association of significantly associated variables were determined by the adjusted odds ratio.

#### Qualitative data

Verbatim transcription was used to convert the audio file of the qualitative data into a text file in Amharic which was translated into English for analysis. The data were then analyzed using the thematic content analysis method using codes and terms to create themes to identify the barriers to PNC service utilization.

Finally, qualitative and quantitative findings were triangulated and barriers to PNC service utilization were reported with the quotation of the participants’ direct words.

### Ethics

Ethical approval was obtained from the Ethical Review Board of Debre Markos University and was passed through both zone and district health bureaus before the commencement of the study. Written informed consent was obtained from each participant before the interview and the interviews were conducted in a private room to maintain privacy of the study participants. Participants who were unwilling to participate and wanted to withdraw at any step of the interview had the freedom to do so without any restrictions.

## Results

### Quantitative arm–utilization of PNC services

#### Socio-demographic characteristics

A total of 738 women completed the questionnaires, resulting in a response rate of 98.3%. More than half (57.7%) of the participants were in the age group 25–34 years and the minimum and maximum ages were 18 and 46 years, respectively, with a mean (± standard deviation) age of 29.4 (±6). Around one-third (35.8%) of participants did not attend formal schooling and nearly half (48.4%) were housewives in their occupation (Table 1).

**Table 1:** Sociodemographic characteristics of the study participants.

Characteristics	Number	Percentage
**Age in Years**		
Less than 25	132	17.9
25–34	426	57.7
>34	180	24.4
**Religion**		
Christian	525	71.1
Muslim	213	28.9
**Educational Status**		
No formal schooling	264	35.8
Primary	207	28.1
Secondary	141	19.0
Higher	126	17.1
**Occupation**		
Housewife	357	48.4
Merchant	111	15.0
Farmer	114	15.5
Government employee	111	15.0
Daily laborer and private workers	45	6.1
**Marital Status**		
Married	654	88.6
Not married	27	3.7
Divorced	45	6.1
Widowed	12	1.6
**Husband’s Age**		
Less than 25	18	2.8
25–34	306	47.2
>34	324	50.0
**Husband’s Educational Status**		
No formal schooling	186	28.4
Primary	201	30.7
Secondary	99	15.2
Higher	168	25.7
**Husband’s Occupation**		
Merchant	237	36.1
Farmer	201	30.6
Governmental Employee	165	25.1
Daily laborer and private workers	54	8.2
**Number of family Members**		
2–4	408	55.3
5–7	246	33.3
>8	84	11.34
**Wealth Index**		
Poor	246	33.3
Moderate	246	33.3
Wealthy	246	33.3

#### Obstetric and behavioral characteristics

One-third (33.3%) of the respondents were para one in terms of their parity. Nearly one in ten participants had antenatal care (ANC) visits with nearly half (53.6%) receiving at least four visits. Around one in ten (10.2%) respondents utilized a maternity waiting home for their most recent childbirth. About three-quarters of the respondents were aware of PNC service utilization and more than half (57.3%) were unaware of postpartum maternal complications (Table 2).

**Table 2:** Obstetric and behavioral characteristics of participants.

Characteristics	Number	Percentage
**Parity**		
1	246	33.3
2–4	360	48. 8
>5	132	17.9
**Antenatal care Visit**		
Yes	666	90.2
No	72	9.8
Number of antenatal care Visits		
1	21	3.2
2	81	12.2
3	207	31.0
>4	357	53.6
**Maternity Waiting Home Use**		
Yes	75	10.2
No	663	89.8
**Place of Delivery**		
Home	39	5.3
Health Post	6	0.8
Health Center	393	53.3
Hospital	300	40.6
**Obstetric Complications**		
Yes	120	16.3
No	618	83.7
**Mode of delivery**		
Normal	537	72.8
Instrumental	153	20.7
Cesarean section	48	6.5
**Birth outcome**		
Alive	708	95.9
Stillborn	30	4.1
**Awareness of Postnatal care**		
Yes	564	76.4
No	174	23.6
**Knowledge of postpartum maternal complications**		
Yes	423	57.3
No	315	42.7
**Member of Women Health Development Army**		
Yes	276	37.4
No	462	62.6
**Partner supportive of Postnatal Care**		
Unsupportive	60	8.7
Neutral	204	29.4
Supportive	429	61.9
**Distance to a health facility**		
Distant	207	28.1
Moderate	318	43.1
Near	213	28.8
**Use of Traditional or Religious practices**		
Yes	168	22.8
No	570	77.2

#### PNC service utilization

**Fig 1:**
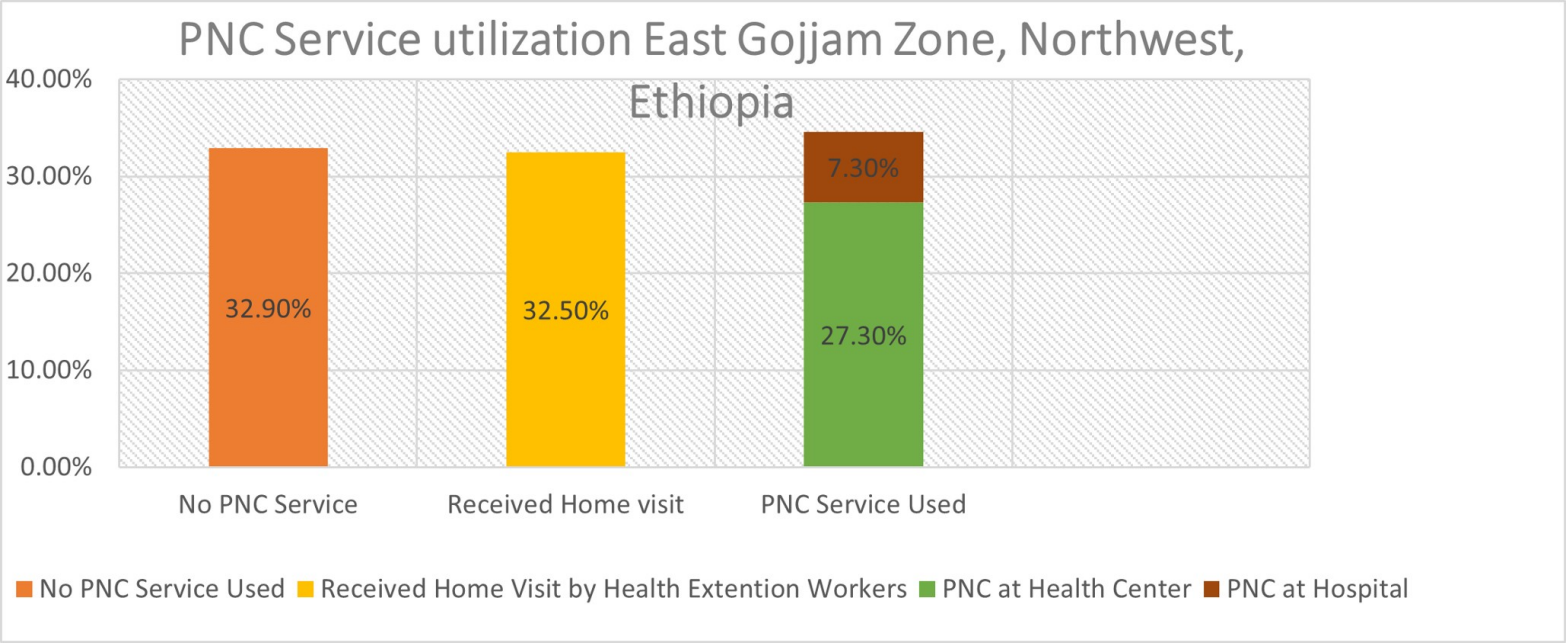
PNC Service utilization in East Gojjam Zone, Northwest Ethiopia.

As indicated in Fig 1, nearly one-third (34.6%) of the study participants visited a health facility for PNC service utilization and 32.5% of the participants were visited by health extension workers at home.

#### Factors associated with PNC service utilization

Factors associated with PNC service utilization were identified by binary logistic regression analysis. All independent variables were explored for their eligibility for logistic regression and eligible variables were then used for the analysis. Variables with a *p*-value <0.2 in the bivariate logistic regression were selected for multivariable logistic regression. A *p*-value <0.05 was used to declare a significant association in the multivariable binary logistic regression. The results indicated that participants’ age, religion, maternity waiting home utilization, mode of birth, awareness of PNC, partner support of maternal and child health MCH service utilization, and distance to a health facility showed significant association with PNC service utilization.

The odds of using PNC services were greater in women who aged 25–34 years (AOR = 2.54, 95%CI = 1.26, 5.15) and those who used maternity waiting home (AOR = 2.80; 95%CI = 1.33, 5.86) (p<0.05). In contrast, Muslim women [AOR = 0.40; 95%CI = 0.22, 0.73), women who had normal (AOR = 0.24; 95%CI = 0.09, 0.65) or instrumental birth (AOR = 0.10; 95%CI = 0.04, 0.30), those who were not aware of the PNC services (AOR = 0.12; 95%CI = 0.05, 0.30) and women whose partners were not supportive of the use of MCH services (AOR = 0.18; 95%CI = 0.05, 0.74) were less likely to use PNC services (p>0.05) (Table 3).

**Table 3:** Factors associated with postnatal care service utilization.

Characteristics	PNC^¶^ Use		COR (95% CI)	AOR (95% CI)
	No	Yes		
**Age (years)**				
<25	102	30	1.31(0.75, 2.28)	0.93(0.38, 2.27)
25–34	234	192	3.66(2.39, 5.58) **	2.54(1.26, 5.15) **
>35	147	33	1.00	1.00
**Religion**				
Christian	306	219	1.00	1.00
Muslim	177	36	0.28(0.19, 0.42) **	0.40(0.22, 0.73) **
**Educational Status**				
No formal schooling	198	66	1.00	1.00
Primary	144	63	1.31(0.874, 1.97)	0.83(0.41, 1.70)
Secondary	93	48	1.55(0.99, 2.43)	0.86(0.35, 2.10)
Higher	48	78	4.87(3.09, 7.68) **	2.05(0.73, 5.77)
**Husbands’ Educational Status**				
No formal schooling	135	51	1.00	1.00
Primary	150	51	0.90(0.57, 1.41)	0.71(0.33, 1.50)
Secondary	60	39	1.72(1.03, 2.88) *	0.46(0.18, 1.18)
Higher	75	93	3.28(2.11, 5.11) **	0.71(0.26, 1.95)
**Family Size**				
2–4	240	168	1.00	1.00
5–7	171	75	0.63(0.43, 0.88) **	0.76(0.43, 1.33)
>8	72	12	0.24(0.13, 0.45) **	1.64(0.42, 6.32)
**Parity**				
1	144	102	1.00	1.00
2–4	222	138	0.88(0.63, 1.22)	0.91(0.49, 1.66)
>5	117	15	0.18(0.10, 0.33) **	0.42(0.11, 1.61)
**Maternity Waiting Home Use**				
Yes	39	36	1.87(1.16, 3.03) **	2.80(1.33, 5.86) **
No	444	219	1.00	1.00
**Mode of delivery**				
Normal	351	186	.65(0.43, 0.97) *	0.24(0.09, 0.65) **
Instrumental	114	39	3.15(1.71, 5.79) **	0.10 (0.04, 0.30) **
Cesarean section	18	30	1.00	1.00
**Awareness of PNC**				
Yes	318	246	1.00	1.00
No	165	9	0.07(0.04, 0.14) **	0.12(0.05, 0.30) **
**Knowledgeable of postpartum complications**				
Yes	183	132	1.76(1.29, 2.39) **	1.40(0.88, 2.22)
No	300	123	1.00	1.00
**Partner supportive of MCH** ^¶¶^ **services**				
Unsupportive	51	9	0.18(0.09, 0.37) **	0.18(0.05, 0.74) *
Neutral	183	21	0.12(0.07, 0.19) **	0.17(0.09, 0.32) **
Supportive	216	213	1.00	1.00
**Distance to a health facility**				
Distant	165	42	0.31(0.20, 0.48) **	0.24(0.12, 0.48) **
Moderate	201	117	0.71(0.50, 1.01)	0.69(0.42, 1.15)
Near	117	96	1.00	1.00

¶ PNC (Postnatal Care);

¶¶ MCH (Maternal and Child Health);

*P-Value < 0.05;

** P-Value < 0.01.

### Qualitative arm—barriers to postnatal care service utilization

We analyzed the qualitative data obtained from three focused group discussions and nine in-depth interviews to identify the barriers to PNC service utilization. The analysis yielded two main themes including ‘knowledge’ and ‘access’. The theme knowledge encompassed two sub-themes as lack of awareness and traditional beliefs and religious practices. Three sub-themes were identified under access as distance and transportation, environmental exposure, and waiting time.

#### Knowledge

*a*. *Lack of awareness*. During both focused group discussions and in-depth interviews, participants indicated a lack of awareness of the necessity of visiting health facilities PNC services and mentioned that one of the reasons for not seeing health care providers in health facilities after giving birth is unawareness of the service. For example, one woman said:

“…*I am not aware of the necessity to visit the facility after giving birth and I haven’t ever used it*.*”*

The issue of lack of awareness was shown to be widespread in the community and the participants believed that only those who become sick need to use the service. They expressed their concerns about a lack of education from the health care providers and believed that it is their responsibility to inform the public of such services. For instance, one woman said:

*“The majority of people in the community don’t have the awareness of the service after birth*. *Thus*, *health care providers have to create awareness and put pressure to use the service*.*”*

The issue of lack of awareness was also raised by health care providers. They mentioned that the benefits of using the PNC services are not properly understood by the women, and many of them expect a home visit rather than attending the center. For example, one facility leader said:

*“In the community*, *there is poor recognition for the advantage of the services after giving birth*.*”*

Another health extension worker mentioned:

*“Since we usually visit delivered women at their home*, *they assume that they shouldn’t come to the health facility waiting for our visit to their home*.*”*

*b*. *Traditional beliefs and religious practices*. Some women reported that they have been forbidden by traditional beliefs and religious practices in the community from leaving the home after birth, visiting PNC services in health facilities or even attending the court in case of a legal issue. The women mentioned that many family members do not allow a woman who has recently given birth to leave home because of the potential risk to the mother and/or her newborn, as they are “prone to the evil of Satan”. Both Christian and Muslim women reported such beliefs and practices, however, in the Muslim religion the restriction was reported to be longer in duration (45 days, compared to 10 days in the Christian religion, as mentioned by some participants). For example, one woman said:

*“Within 10 days after childbirth*, *women are not allowed to go out of home and move freely in the day time*. *Even they are allowed to go-to toilet in the morning and evening only*.*”*

Another woman said:

*“A woman is allowed to ambulate only after 10 days if she has given birth to a male child and 15 days after having a female child for showering purpose by holy water*.*”*

The issue of traditional beliefs and religious practices hindering women’s attendance at the PNC services in health facilities was also raised by health care workers. For instance, one health extension worker commented:

“… *among the factors that hinder seeking facility visits after birth there is a religious restriction to go out of a home within 10 days and 40 days*.*”*

#### Access

*a*. *Distance and transportation*. Some participants mentioned distance and transportation-related problems as barriers to returning to the health facility for PNC services. Some women mentioned that stakeholder organizations are focused on obtaining transportation for childbirth services, but are not concerned about this for PNC services, despite it being a major issue. District health officers reorganize ambulances to take laboring mothers to a health facility, but they do not do so for PNC services. In the rainy season, the roads get worse and transportation becomes even more difficult. For example, one woman said:

*“We use facility ambulances to go to health facilities for childbirth only*. *After giving birth we are obliged to use our means of transport*. *In the presence of transport means like Bajaj* [a type of vehicle that have three tyers and able to contain one to five people at a time] *we use it but if we are not able to access*, *we use cart or foot walk to come back to the health facility which makes it very hard for a newly delivered mother to seek services*.*”*

Concerns related to distance and transportation were also raised by the health care workers. They reported that this has caused access issue not only for the childbearing women but also for the health care workers and ambulances. For example, one health extension worker said:

*“The presence of mud in the rainy season makes hard to come for postnatal care services in the facilities*. *Even it is extremely hard for us to go home to home visits*. *For this reason*, *we hardly offer the service in such a season*.*”*

*b*. *Environmental exposure*. Environmental conditions, mainly sunlight and wind, also played a key role in reducing the uptake of PNC services. Women explained that their body becomes more susceptible to the weather after giving birth, making it difficult to seek PNC services. One woman commented:

*“After giving birth*, *our body is extremely fragile to be exposed to wind*. *We are not also able to resist sunlight*. *We have also smelling discharge*. *For these reasons*, *we prefer to stay at home for days and weeks*.*”*

Another woman reported:

“*While walking in the day time we have to have an umbrella to protect the exposure to the sunlight which needs an accompanier*. *So*, *it is difficult to visit the facility alone*, *after giving birth*.*”*

*c*. *Waiting time*. A few women mentioned that the length of time they have to spend in the health facility waiting to receive care makes them reluctant to attend. Having a newborn with them along with other requirements of postnatal period were reported to make it difficult for the women to visits the center for postnatal services. For instance, one woman expressed her concern of exposing her newborn

*“Women who come to health facilities with a newly born child wait their turn with other women to receive health services*. *Some of them also don’t want to expose their child in front of multiple persons including health care providers*.*”*

## Discussion

This study was conducted to assess postnatal care service utilization and its barriers through the application of a mixed-method, cross-sectional study design in East Gojjam Zone, Northwest Ethiopia. The study determined that 34.6% [95% CI: 0.311, 0.381] of women in this zone visited health facilities for PNC services. Quantitative analysis of women’s age, religion, maternal waiting home utilization, mode of birth, PNC awareness, partner support and distance to health facilities indicated that these were factors associated with PNC service utilization. The analysis also indicated that lack of awareness, traditional beliefs and cultural norms, religious practices, distance, and transportation-related problems, fear of exposure to sunlight and wind, and long waiting times in health facilities were barriers to accessing PNC services.

The utilization of PNC services determined here is lower than those found in Nepal (Demographic Health Survey) [17], India [18], Tanzania [19], Malawi [20], Halaba Kulito town (Southern Ethiopia) [21], Addis Abeba (Ethiopia) [22] and Debre Tabor (Ethiopia) [23]. The discrepancy is most likely due to the study populations and different locations. Specifically, the studies conducted in Ethiopia were mainly confined to urban residents whereas we also included rural residents. However, the utilization of PNC services determined in our study is higher compared to studies conducted in Nepal [24], Nigeria (DHS) [25], northern Nigeria [26], Rwanda (DHS) [27], Ethiopia (analysis on EDHS 2011 [28], EDHS 2016 [12]) and Northwest Ethiopia [29]. The difference might be attributable to the study location and timing, and population variations. The 2016 EDHS reported PNC visits sought by mothers within two days after birth whereas our study considered PNC services received within forty-two days after birth. The findings of our study are consistent with those of studies in northern Ethiopia [30], Wolaita Zone (Southern Ethiopia) [31], and Debre Markos town [13].

Our study showed that women aged 25–34 years were 2.5-fold more likely to utilize PNC services than women aged 35 and above. Likewise, various studies conducted in different parts of the globe report that younger women utilize PNC services to a greater extent than their older counterparts [21, 24, 28, 29]. This could be because younger women have a smaller workload and have more time to utilize maternal health care services as compared to older women [31].

Muslim women were 60% less likely to utilize PNC services as compared to Christian women. This is highly likely due to differences in religious practices. For example, Muslim women are restricted to their homes up to 45 days after birth, whereas Christian women are allowed to leave their homes 10–15 days following childbirth.

Women who used maternal waiting home services for their most recent childbirth were 2.8-fold more likely to use PNC services compared to those who did not use waiting home services. The positive association between maternity waiting home utilization and PNC service utilization might be because women in MWHs are made aware of PNC services during their stay in the health facility for the service. Women who were unaware of PNC services were 88% less likely to utilize these services compared to their informed peers. This result is consistent with those from studies conducted in northern Ethiopia, Addis Abeba (Ethiopia) and Wolaita zone (southern Ethiopia) [19, 27, 28]. The qualitative data from our study also support these findings. For example, some participants of the qualitative study perceived that health facility visits after birth were limited to women with health problems.

Women who gave birth normally or with instrumental assistance were 76% and 90% less likely to utilize PNC services, respectively, as compared to women who gave birth through cesarean section. This result is consistent with that found in a study conducted in Debre Markos, Northwest Ethiopia [13]. This might be linked to a fear of complications for women with a cesarean section, who also need close follow-up to avoid infection. It is also possible that women who gave birth normally had less awareness of the benefits of PNC services.

Women whose partners were not supportive of MCH services use were 82% less likely to use PNC services than those with supportive partners. A similar association was reported in studies conducted in Jabitenan district, Northwest Ethiopia, Wolaita zone, Southern Ethiopia, and Addis Abeba, Ethiopia [19, 26, 28]. Studies show that support by a male partner plays a key role in maternal health care service utilization [32].

Women who lived far from the health facility were 76% less likely to use PNC services as compared to women who lived close by. This finding agrees with that of studies conducted in Nepal and Nigeria [21, 31, 33] and is related to the importance of easily accessible health facilities for maternal health care service utilization [34–36]. The findings of our qualitative study also showed that unavailability of transportation and inconvenient geographical and seasonal conditions restricted the uptake of health care services following childbirth.

Traditional beliefs also played a role in hindering PNC service utilization as explored by the qualitative findings. Environmental factors, specifically fear of exposure to sunlight and wind, were among the barriers mentioned by the qualitative study participants. Women’s bodies undergo physiological changes to allow them to resume their pre-pregnancy state which results in discomfort and makes them susceptible to the extremity of weather changes [37]. The time that women spent in health facilities and the way that the health care providers approached them also contributed to PNC service utilization, as explained by in-depth interview participants.

### Strength and limitations

The strength of our study is the use of a mixed method design which assisted us to have a better understanding of the factors affecting the PNC service utilization and barriers of accessing the service while learning from the participants’ experiences and point of view. Nevertheless, the use of a cross-sectional study design made it impossible to determine a cause and effect association between the factors and PNC service utilizations. Since the study gathered the data from women who gave birth within one year prior to the study period, recall bias could be the other limitation.

### Recommendations

Health care providers should promote PNC service utilization through health education and offer service in culturally sensitive way. The governmental and non-governmental stakeholders should rearrange transportations for PNC service users and work on solving infrastructure related problems mainly constructing roads and accessing the services for women through establishing health care institutions. Finally, the researchers have to further investigate the determinants of PNC service utilization through application of advanced study designs that can detect cause and effect relationship

## Conclusion

This study revealed low utilization of PNC services in East Gojjam Zone, northwest Ethiopia. Factors quantitatively associated with PNC service utilization were age, religion, use of maternity waiting homes, mode of birth, lack of awareness of PNC and distance from the health facility. Qualitatively, lack of awareness of PNC, traditional beliefs, religious practices, distance and transportation-related problems, fear of exposure to sunlight and wind, and long waiting times in health facilities were identified as barriers to PNC service utilization.

## Supporting information

S1 Data (DTA)
